# Self-Regulation and Mathematics Performance in German and Iranian Students of More and Less Math-Related Fields of Study

**DOI:** 10.3389/fpsyg.2020.489371

**Published:** 2020-10-30

**Authors:** Parvin Nemati, Caterina Gawrilow, Hans-Christoph Nuerk, Jan Kühnhausen

**Affiliations:** ^1^Department of Psychology, University of Tübingen, Tübingen, Germany; ^2^LEAD Graduate School & Research Network, University of Tübingen, Tübingen, Germany; ^3^Leibniz-Institut für Wissensmedien, Tübingen, Germany; ^4^Department for Child and Adolescent Psychiatry, Psychosomatics and Psychotherapy, University of Tübingen, Tübingen, Germany

**Keywords:** self-regulation, mathematics, cross-culture, field of study, multiplication

## Abstract

Self-regulation is a multidimensional construct that is positively related to academic achievement, such as successful mathematics performance. However, this relation of self-regulation and mathematics performance has mainly been investigated in Western countries with similar cultural contexts, although self-regulation is assumed to be context-sensitive. Therefore, the present study investigated the relation of self-regulation and mathematics performance across two different countries (Germany vs. Iran) in college students. The relation of self-regulation and mathematics performance was expected to be weaker in students of math-related fields, such as Engineering/Informatics, as they are assumed to need less self-regulation to solve the mathematics problems than students of less math-related fields, such as Human Sciences. In total, 122 undergraduate students (German = 60; Iranian = 62) of Human Sciences or Engineering/Informatics participated in this study. We measured self-regulation with the Brief Self-Control Scale (Tangney et al., 2004) and mathematics performance with a complex multiplication test. Results showed that self-regulation did not predict multiplication performance in German or Iranian students, in general. However, when the field of study was considered, self-regulation predicted multiplication performance in the subgroup of German and Iranian students studying Human Sciences within each country. We conclude that cultural context does not seem to play a dominant role in moderating the relation between self-regulation and math performance, however, field of study and more generally familiarity with math may be an important factor to consider in single or cross-cultural studies.

## Introduction

Self-regulation is defined as the ability to control one’s thoughts, behaviors, or emotions, and enables individuals to adapt their behaviors in accordance with the demands of a situation (e.g., Baumeister and Vohs, 2007; Blair and Ursache, 2011). It includes abilities such as maintaining attention and inhibiting irrelevant information in learning situations, which provides an important foundation for successful academic outcomes (e.g., McClelland and Cameron, 2011). A large body of research connects self-regulation with different academic achievements, such as successful mathematics performance (e.g., Zimmerman, 1990; Bull and Scerif, 2001; Camahalan, 2006; Fuchs et al., 2006; Blair and Razza, 2007; Labuhn et al., 2010; McClelland et al., 2010; Otts, 2010; von Suchodoletz and Gunzenhauser, 2013; Gawrilow et al., 2014). For instance, college students with better self-regulation abilities measured by self-reports have been shown to respond more rapidly in mathematics tasks, which could be because of their enhanced ability to ignore distracting thoughts and concentrate on the task (Nemati et al., 2017). In contrast, students without adequate self-regulatory skills are more likely to experience difficulties in mathematics performance. For example, students who struggle with self-regulation, such as students with attention deficit/hyperactivity disorder (ADHD) have more difficulty with mathematics at school (e.g., Frazier et al., 2007; Zentall, 2007).

Previous studies have indicated that self-regulation contributes to mathematical performance by suppressing distracting thoughts or information whilst mathematics problems are solved (e.g., Gawrilow et al., 2011; Nemati et al., 2017), and through different cognitive components of self-regulation such as inhibitory control (e.g., Hofmann et al., 2011; McClelland and Cameron, 2011). For instance, solving complex multiplication problems requires ignoring distracting thoughts to remain focused on the task and selecting the correct solutions while suppressing alternative ones (e.g., neighboring solutions in the multiplication table) that can interfere with the retrieval of a desired solution (e.g., “42” can interfere with retrieving the answer to “6 × 8”; cf. Domahs et al., 2006, 2007).

However, the relation of self-regulation and mathematics performance might vary across different contexts. Recent studies demonstrated that self-regulation is a context-specific construct (e.g., Keller et al., 2004; von Suchodoletz et al., 2015; Lamm et al., 2018), suggesting that context can influence self-regulation displayed in different situations. For instance, the different parenting styles of European American and Puerto Rican mothers resulted in different patterns of self-regulation development during childhood (Carlson and Harwood, 2003): in the European American context, mothers expected their children to alter their behavior to match their individual goals, while Puerto Rican mothers asked their children to adjust their behavior in accordance to the society.

These findings are in line with the theoretical framework of Markus and Kitayama (1991), suggesting *independent and interdependent* contexts, which can influence self-regulation. Independent contexts focus on autonomy and individual goals, whereas interdependent contexts are associated with being in harmony with the group and the community goals. Accordingly, self-regulation processes in an independent context are directed toward influencing the environment and other people in line with an individual’s goals, while in interdependent contexts they focus on adjusting one’s behavior to the expectations of others to maintain fit with the group (Trommsdorff, 2009). For instance, the results of a recent cross-cultural study (Lamm et al., 2018) revealed that the development of self-regulation and self-regulatory strategies used by children can be different in independent and interdependent contexts. They showed that while German mothers emphasized autonomy and individual goals of their children, Cameroonian mothers expected their children to behave in harmony with society. Thus, German children’s self-regulation was motivated by a different goal (i.e., autonomy in Germany vs. parents’ expectations and group harmony in Cameroon) and for the same reason, German children might have used different self-regulatory strategies than their Cameroonian peers to do the self-regulation task.

Previous studies have showed that independent contexts are a core characteristic of Central European and North American countries, while interdependent contexts prevail in Asian and Latin American countries (e.g., Higgins et al., 2008; Trommsdorff, 2009). In the same line, individualism and autonomy are valued in Germany, while collectivism and group harmony are respected in Iran (Hofstede, 1980). Therefore, Germany and Iran provide two different contexts with distinct environmental characteristics that can affect self-regulation and its correlates.

However, although self-regulation has been frequently shown to have a context-sensitive nature (Trommsdorff, 2009; see also the review by Jaramillo et al., 2017), less is known about the relation of self-regulation and academic achievement, such as mathematics performance, across different countries and the existing results in children are rather scarce and heterogeneous. On the one hand, results of a cross-cultural study in preschool children demonstrated that the associations between different components of self-regulation and mathematics performance were largely similar between Chinese and North American children (Lan et al., 2011). They discussed that their finding might be due to the similarities in the associations between different cognitive components of self-regulation in distinct contexts. On the other hand, results of a longitudinal study investigating the application of self-regulatory strategies in educational settings, showed that many of the self-regulatory strategies used by Italian students did not predict the academic achievement as they did in American students (Nota et al., 2004). Researchers examined the self-regulatory strategies adopted by Italian students during the final year of high school and their academic achievement in pursuing further education at the University and compared their results with previous studies in American students. In the same vein, but in contrast to previous studies in Western countries, results of another study on Chinese students revealed no relationship between self-regulation and mathematics achievement in Chinese high school students (Rao et al., 2000). The authors suggested that self-regulatory strategies motivated by Chinese attitudes toward academic achievement and parents’ expectations could not predict mathematics performance in Chinese high school students. Therefore, self-regulatory strategies adopted by students might not be equally important in predicting mathematics achievements across different countries. Altogether, it seems that independent and interdependent contexts can potentially impact the relation of self-regulation and mathematics performance. Furthermore, differences in self-regulatory skills across different countries can persist in adolescence (e.g., Ellefson et al., 2017), suggesting that context may influence self-regulation and its subsequent relationship with future academic, in particular, mathematics performance. Therefore, the aim of the present study was to examine whether the relation of self-regulation and mathematics performance varies between German and Iranian college students.

Additionally, field of study was considered as another context beside the country that could influence the relationship between self-regulation and mathematics performance in college students. It has been shown that individuals need more self-regulation when doing difficult tasks (e.g., Kanfer and Ackerman, 1989; Steele-Johnson et al., 2000) and solving mathematics problems might be less difficult for students of math-related fields, as compared to students of less math-related fields. Accordingly, context of field of study might affect the relationship between self-regulation and mathematics performance: the relationship between self-regulation and mathematics performance was expected to be weaker in students of math-related fields, such as Engineering/Informatics, as they are assumed to need less self-regulation to solve the mathematics problems than students of less math-related fields, such as Human Sciences. Therefore, the context-effect of field of study was taken into account in the present study as it can influence the students’ mathematics performance and hence alter its relationship with self-regulation.

To sum up, in the present study, we hypothesized that the relation of self-regulation and mathematics performance differs in German and Iranian college students as independent and interdependent contexts can differentially affect self-regulation and its correlates. Furthermore, as the second hypothesis, we expected that the relation of self-regulation and mathematics performance is weaker in students of math-related fields, such as Engineering/Informatics, than in students of less math-related fields, such as Human Sciences, because less self-regulation is needed for doing relatively less difficult tasks.

## Materials and Methods

### Participants

Participants were 60 German^1^ (41 females, age: *M* = 21.15 years, *SD* = 1.15) and 62 Iranian (28 females, age: *M* = 20.53 years, *SD* = 1.18) undergraduate students. The German participants were recruited from the University of Tübingen in south Germany and Iranian participants were from the University of Tehran, Iran. All participants were native speakers with no immigration backgrounds. The entire data of the participants were analyzed anonymized (i.e., using personal codes instead of names). Detailed characteristics of both German and Iranian students are depicted in Table 1.

**TABLE 1:** Descriptive and test statistics of background characteristics and study measurements.

**Variable**	**German**			**Iranian**			**Diff**
	***n***	***M* (*SD*)**	***K-S^a^***	***n***	***M* (*SD*)**	***K-S^a^***	***P***
Age (years)	60	21.15 (1.15)	**<0.001**	62	20.53 (1.18)	**<0.001**	**0.005**^b^
Gender, female	41			28			**0.011**^c^
Field of study							
Human Sciences	40			32			
Engineering/Informatics	20			30			
Math self-concept	60	2.72 (0.80)	**<0.001**	62	2.58 (1.25)	**<0.001**	0.738^b^
Expectancy of success	60	2.88 (0.64)	**<0.001**	62	3.26 (0.92)	**<0.001**	**0.001**^b^
Self-regulation	60	40.92 (8.53)	200	62	42.56 (6.40)	200	0.229^d^
Multiplication performance							
ER	60	0.18 (0.10)	0.077	62	0.19 (0.10)	**0.001**	**<0.001**^b^
RT(s)	60	3.05 (0.54)	200	62	2.59 (0.59)	0.073	**<0.001**^d^

*^*a*^Kolmogorov–Smirnov *p*-values, ^*b*^Mann–Whitney *U* Test,^*c*^Fisher’s Exact Test, ^*d*^*t*-test. Bold *p*-values depict *p* < 0.05.*

### Measures

#### Background Characteristics

Background characteristics, consisting of field of study, math score in the University entrance exam, math self-concept, expectancy of success, and demographics of the participants (gender, age, nationality, citizenship, mother tongue, language spoken at home) were collected with a background questionnaire. The questions of the background questionnaire, except the questions of math self-concept, were developed by the authors. Math self-concept was assessed by four questions (e.g., “I am good at mathematics.”) based on the SDQ (Self Description Questionnaire) III (Marsh, 1992; German translation: Schwanzer et al., 2005).

#### Self-Regulation

Participants’ self-regulation was assessed by using self-reports. Participants were asked to fill out the Brief Self-Control Scale (BSCS; Tangney et al., 2004; German translation: Bertrams and Dickhäuser, 2009). The German translation of the BSCS (Bertrams and Dickhäuser, 2009) was used in Germany. The original English version of the BSCS was translated into Farsi by two bilingual Ph.D. students from the Psychology field and one bilingual Ph.D. student from outside the field using a well-established method of forward- and backward-translations, following the guidelines from the World Health Organization, 2015).

The BSCS consists of 13 items targeting thought control, impulsive response control, action persistence, and action monitoring (e.g., “I wish I had more self-discipline.”). The response format was a 5-point Likert-type scale ranging from 1 (*completely true)* to 5 (*completely untrue*). Nine items were reverse-coded and the total score was the sum of the responses of all items, with higher sum scores representing more self-regulation. In the present study, the questionnaire showed sufficient internal consistency (in German students: Cronbach’s α = 0.84; in Iranian students: Cronbach’s α = 0.70).

#### Mathematics Performance

Mathematics performance was assessed by using the complex multiplication test, consisting of 48 complex multiplication problems. The complex multiplication problems entailed one-digit times two-digit problems with two-digit solutions (e.g., 4 × 19 = 76; for further details, see Nemati et al., 2017). The complex multiplication problems and their solutions were presented in a computerized verification task, programmed with the PsychoPy software (Peirce, 2009). Half of the presented solutions were correct, and the other half were incorrect. The task started with eight practice trials. All trials were presented in the center of the screen in a fixed order. The problems and their solutions were presented at the same time after the 500 ms fixation point and remained on the screen until a response was given by the participant, or 6000 ms had passed. Participants responded by pressing the green or red keys (*L* and *A* on a German keyboard) for correct and incorrect solutions, respectively. The response keys were counterbalanced across participants. Except for practice trials, all trials were presented without feedback.

### Procedure

All German participants were recruited through e-mail to students and staff of the University of Tübingen and in-person contact. All Iranian participants were recruited through flyers and in-person contact in the University of Tehran. The study on German students of Human Sciences was part of a larger project consisting of two testing sessions, each lasting about 2 h, aimed at examining the effects of self-regulatory training on the academic performance of young adults. For their participation, German students of Human Sciences received either course credits or 8 Euro per hour. German students of Engineering/Informatics as well as all the Iranian participants were offered chocolates for their approximately 10 min participation in the study consisting of filling out the background and BSCS questionnaires plus answering the complex multiplication test. First, all participants received detailed information about the study and later gave their written informed consent to participate in the study. The testing session took place in a laboratory in Germany or in an empty classroom of the University of Tehran in Iran. For the variables reported here, each participant was tested individually in a single session. First, all participants were asked to fill out the computerized version of the questionnaires consisting of background questionnaire and BSCS items, which lasted roughly 5 min. Subsequently, they were asked to perform the computerized complex multiplication task, which lasted about 5 min. Participants received a detailed written instruction emphasizing the importance of both speed and accuracy of the responses in the complex multiplication task.

### Analysis

#### Data Preparation

In the present study, better performance in the complex multiplication test was indicated by shorter response times (RTs) and lower error rates (ERs). Multiplication RTs of the participants were defined by the time intervals between the presentation of the multiplication problems on the screen and the responses of the participants, measured by pressing the keys of the computer keyboard. Only RTs of correct responses were considered in the analyses. Moreover, RTs shorter than 200 ms were excluded, and subsequently RTs which were more or less than ± 3 SD around the individual mean were excluded continually until no more outliers remained (see: Nuerk et al., 2001, and follow-up papers for the same method). Accordingly, about 0.1% of the RTs of the German students and 0.2% of the RTs of the Iranian students were excluded. Furthermore, in Germany, two multiplication trials, which were planned to presented with presented with correct solutions, were mistakenly presented with incorrect solutions. Therefore, to keep the match of trials with correct and incorrect solutions, those two trials plus their two equivalent ones with incorrect solutions were excluded from the data of the German students.

Multiplication ERs of the participants were defined as the proportion of incorrect responses. ERs are briefly reported in the descriptive statistics (Table 1) but not considered for the further statistical analyses because the performance of German and Iranian students indicated a ceiling effect, as they made few errors in the complex multiplication task (see Table 1 and Appendix A). Finding a ceiling effect in multiplication performance is not surprising as highly educated adults often perform at above-average levels in mathematics tasks (e.g., Siegler and Opfer, 2003; Karolis et al., 2011). Moreover, there were five missing answers in BSCS of two German participants that were replaced by the mean of BSCS answers of the same participants.

#### Relation of Self-Regulation and Multiplication Performance in German and Iranian Students

The first hypothesis of the present research was that the relation of self-regulation and mathematics performance differs in Germany and Iran. First, to test the effect of self-regulation on multiplication performance in German and Iranian students, a separate linear regression analysis was conducted for each subsample (i.e., German students, Iranian students) with self-regulation as predictor and mean multiplication RTs as outcome variable. In the second step, to compare the relation of self-regulation and mathematics performance between German and Iranian students, the linear regression analysis was calculated with self-regulation, country (dummy coded), and the interaction between self-regulation and country as predictors and mean multiplication RTs as the outcome variable.

#### Effect of Field of Study on the Relation of Self-Regulation and Multiplication Performance

The second hypothesis of the present research was that the relation of self-regulation and mathematics performance is weaker in students of Engineering/Informatics. In the first step, four separate linear regression analyses were conducted for each subsample field of study (i.e., German and Iranian students of Human Sciences and Engineering/Informatics) with self-regulation as predictor and mean multiplication RTs as outcome variable. In the second step, to compare the relation of self-regulation and mathematics performance in students of Human Sciences and Engineering/Informatics, the interaction between self-regulation and field of study was tested in a multiple linear regression analysis with self-regulation, field of study (dummy coded), and the interaction between self-regulation and field of study as predictors and mean multiplication RTs as the outcome variable. All continuous variables were standardized and the level of significance was set to α < 0.05 for all analyses.

## Results

### Descriptive Statistics

Descriptive and test statistics for the background characteristics and the study measurements of German and Iranian students are presented in Table 1^2^. In case of non-normally distributed variables (Kolmogorov–Smirnov test *p*-values < 0.05), Mann–Whitney *U* test, and for normally distributed variables *t*-test and Fisher’s Exact Test were used.

German and Iranian students did differ in most of the background characteristics, such as age, *U* = 2.40, *p* = 0.005, gender, *p* = 0.011, Fisher’s Exact Test, and expectancy of success, *U* = 1.25, *p* = 0.001. Although German and Iranian students significantly differed in age and gender (see Table 1), our result was not explained neither by age nor by gender differences between the two countries (see Appendix D).

Additionally, German and Iranian students did differ in their multiplication performance: German students were slower, *t*(120) = −4.46, *p* < 0.001, *d* = 0.81, and Iranian students made more errors, *U* = 2.62, *p* < 0.001. However, German and Iranian students did not differ in math self-concept, *U* = 1.92, *p* = 0.738, and self-regulation, *t*(120) = 1.21, *p* = 0.229, *d* = 0.22.

### Relation of Self-Regulation and Multiplication RT in German and Iranian Students

Regression analysis revealed that self-regulation did not predict multiplication RT neither in German (*b* = −0.25, *t* = −1.10, *p* = 0.051; see Table 2) nor in Iranian students (*b* = −0.09, *t* = −0.72, *p* = 0.473; see Table 2). Moreover, the non-significant interaction indicates that the relation of self-regulation and mathematics performance did not significantly differ between German and Iranian students (*b* = −0.09, *t* = −0.53, *p* = 0.599; see Table 2). The data met the assumptions of collinearity (self-regulation, tolerance = 0.36, *VIF* = 2.75*;* country, tolerance = 0.99, *VIF* = 1.01; self-regulation × country, tolerance = 0.37, *VIF* = 2.73), independent errors (Durbin–Watson value = 1.70), and non-zero variances (self-regulation, variance = 56.80; country, variance = 0.25; self-regulation × country, variance = 0.63) and contained no outliers (*Std. Residual Min* = −2.57, *Std. Residual Max* = 2.71).

**TABLE 2:** Regression analysis predicting multiplication RT from self-regulation in German and Iranian students.

**Predictor**	***b***	***SE(B)***	***t***	***p***
**Model 1**				
Self-regulation in Germans^a^	−0.25	0.13	−1.10	0.051
**Model 2**				
Self-regulation in Iranians^b^	−0.09	0.13	−0.72	0.473
**Model 3**				
Constant	−0.36	0.12	−3.03	**0.003**
Self-regulation	−0.11	0.14	−0.76	0.446
Country	0.72	0.17	4.27	**<0.001**
Self-regulation × country	−0.09	0.17	−0.53	0.599

*^a^n = 60, ^b^n = 62. All variables are standardized and country was dummy coded. Bold *p*-values depict *p* < 0.05.*

### The Effect of Field of Study on the Relation of Self-Regulation and Multiplication RT

**FIGURE 1:**
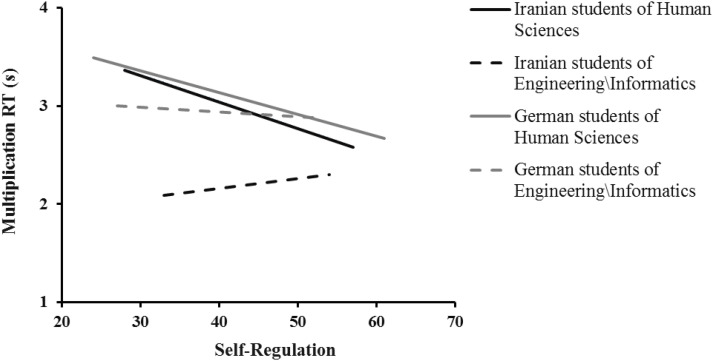
Linear regression trend lines testing the relation of self-regulation as predictor and multiplication RT as the dependent variable in four different subsamples: German students of Human Sciences, German students of Engineering/Informatics, Iranian students of Human Sciences, and Iranian students of Engineering/Informatics.

As shown in Table 3 and Figure 1, there is a significant negative relationship between self-regulation and multiplication RT in German [*b* = −0.35, *t* = −2.26, *p* = 0.029; Model 1: *R*^2^ = 0.12, *F*(1,38) = 5.12, *p* = 0.029] and Iranian [*b* = −0.29, *t* = −2.23, *p* = 0.034; Model 3: *R*^2^ = 0.14, *F*(1,30) = 4.96, *p* = 0.034] students of Human Sciences, but not in German (*b* = −0.07, *t* = −0.34, *p* = 0.736) and Iranian (*b* = 0.12, *t* = 0.80, *p* = 0.428) students of Engineering/Informatics (see Table 3). Similar decreasing trends in Human Sciences showed in Figure 1, indicating the higher the self-regulation the better the students of Human Sciences within each countries performed in the complex multiplication task.

**TABLE 3:** Regression analysis predicting multiplication RT from self-regulation in German and Iranian students of Human Sciences and Engineering/Informatics.

**Predictor**	***b***	***SE(B)***	***t***	***p***
**Model 1**				
Self-regulation of German students of Human Sciences^a^	−0.35	0.15	−2.26	**0.029**
**Model 2**				
Self-regulation of German students of Engineering/Informatics^b^	−0.07	0.22	−0.34	0.736
**Model 3**				
Self-regulation of Iranian students of Human Sciences^c^	−0.29	0.13	−2.23	**0.034**
**Model 4**				
Self-regulation of Iranian students of Engineering/Informatics^d^	0.12	0.13	0.80	0.428
**Model 5**				
Constant	0.38	0.10	3.75	**<0.001**
Self-regulation	−0.30	0.10	−3.01	**0.003**
Field of study	−0.93	0.16	−5.79	**<0.001**
Self-regulation × field of study	0.21	0.16	1.31	0.194

*^a^n = 40, ^b^n = 20, ^c^n = 34, *^*d*^n* = 30. All variables are standardized and country was dummy coded. Bold *p*-values depict *p* < 0.05.*

Moreover, the non-significant interaction indicates that the relation of self-regulation and mathematics performance did not significantly differ between students of Human Sciences and Engineering/Informatics (*b* = 021, *t* = 131, *p* = 0.194; Table 3). The data met the assumptions of collinearity (self-regulation, tolerance = 0.63, *VIF* = 1.60; field of study, tolerance = 0.99, *VIF* = 1.00; self-regulation × field of study, tolerance = 0.63, *VIF* = 1.60), independent errors (Durbin–Watson value = 1.83), and non-zero variances (self-regulation, variance = 56.80; field of study, variance = 0.24; self-regulation × field of study, variance = 0.37) and contained no outliers (*Std. Residual Min* = −2.49, *Std. Residual Max* = 2.35).

## Discussion

The present study investigated whether the relation of self-regulation and mathematics performance differs between students in two different contexts, namely independent and interdependent cultures (i.e., Germany vs. Iran). As the second hypothesis, we expected that the relation of self-regulation and mathematics performance was weaker in students of Engineering/Informatics as compared to students of Human Sciences. Contradictory to our first hypothesis, the relation of self-regulation and mathematics performance did not differ between German and Iranian college students: self-regulation did not predict multiplication RT neither in German nor Iranian students. Moreover, inconsistent with our second hypothesis, the results showed that the relation of self-regulation and mathematics performance did not differ significantly between students studying less math-related fields (i.e., Human Sciences) and students of math-related fields (i.e., Engineering/Informatics) in the whole sample. However, partially in line with our second hypothesis, when the field of study was considered within the countries, self-regulation predicted multiplication RT in those students studying Human Sciences but not in students of Engineering/Informatics within each country. Thus, although the main effect of field of study was not observed regardless of country, the relation of self-regulation and mathematics performance seemed to be descriptively weaker in students of Engineering/Informatics than Human Sciences within each country. This might be because the complex multiplication test within each country seemed to be less difficult for the students of Engineering/Informatics compared to the students of Human Sciences, therefore, these students might need less self-regulation to solve the problems. The complex multiplication test seemed to be less difficult for the students of Engineering/Informatics as they performed better (i.e., they had shorter RT and less ER) than students of Human Sciences in general (see Appendix B). However, this effect was significantly different in Iranians but only descriptively different in Germans (see Appendix B). Moreover, expectancy of success in math was higher in students of Engineering/Informatics than students of Human Sciences (see Appendix B) within each country and significantly correlated with shorter RTs (see Appendix C), suggesting that students of Engineering/Informatics believed in their self-ability to do well in mathematics. Thus, students of Engineering/Informatics within each country might have used less self-regulation while doing complex multiplication test as the test was less difficult for them. This is consistent with previous studies revealing that individuals need more self-regulation while solving challenging tasks (e.g., Ackerman, 1989; Kanfer and Ackerman, 1989; Steele-Johnson et al., 2000). For instance, it has been shown that task difficulty can moderate the effect of self-regulation on performance (Steele-Johnson et al., 2000). The authors found that when the cognitive load of the task is high, individuals have to decide how to allocate their limited attentional resources to the task, therefore, they are in need of more self-regulation.

Taken together, the results showed that the relation of self-regulation and mathematics performance did not differ between German and Iranian college students. Furthermore, we observed this similarity not only in the context of country but also in the context of field of study, which is further supported by the fact that when only the students of Human Sciences are compared, the association between self-regulation and mathematics is similar in both countries (Appendix E). This finding is in line with a cross-cultural study by Lan et al. (2011), described earlier, that assessed the cognitive components of self-regulation, such as inhibition and attentional control, and examined their associations with simple and complex mathematics performances in Chinese and North American children. Their results demonstrated that the relation of different cognitive aspects of self-regulation and both simple and complex mathematics performance are similar in Chinese and North American children. The authors argued that the neurobiological and genetic factors which determine the strength of associations between various components of self-regulation may be similar in distinct contexts, therefore, their subsequent contribution to academic performance is also more likely to be consistent across countries. However, Chinese children outperformed North American children in some of the self-regulation tasks such as inhibition and attentional control. The authors ascribed these performance differences in self-regulation tasks to variances in specific cultural practices in educational settings during kindergarten and primary school. For instance, it has been shown that Asian children receive more intensive practice in controlling their attention and behavior in kindergarten or the classroom than North American children (e.g., Chen et al., 1998; Kwon, 2004; Lan et al., 2009). Therefore, it seems that although different aspects of self-regulation may be learned and used differently in interdependent and independent countries, their interrelations with each other and their association with mathematics performance remains similar. This interpretation is also in line with the idea that both independent and interdependent systems exist and are essential in each country, but there might be differences among the countries in the strength of their application (e.g., Harwood et al., 2001; Leyendecker et al., 2002; Jing-Schmidt, 2014). In the same vein, both independent and interdependent self-regulation processes may exist in Germany and Iran to different degrees, but this may not significantly influence their level of contribution to the mathematics performance.

However, our finding is in contrast with previous studies, connecting the academic achievement gap between students from different countries to the effect of cultural context on self-regulation. For instance, in a longitudinal study by Nota et al. (2004) which was explained earlier, many of the self-regulatory strategies that predicted academic achievement in American students did not directly predict academic achievement in Italian students. However, compared to the study by Nota et al. (2004), the effect of various self-regulatory strategies was not investigated in the present study and contexts as well as measures of self-regulation and academic achievement differ from their study. Another important reason why, unlike our study, they found differences in the relation of self-regulation and academic achievement across two countries, might be the effect of samples: Italian students were high achievers who are more likely to self-regulate than typical populations of students and in this sense differed from the American students or from German and Iranian students in our research.

Altogether, cultural context did not seem to play a dominant role in moderating the relation between self-regulation and math performance in the present study. However, with regard to the confounding effect of field of study within each country on the predictive validity of self-regulation, careful sample selection considering field of study of students is recommended for future research examining the relation of self-regulation and mathematics performance.

### Limitations

The current research has some limitations worth noting. First, there might be structural and cultural variations in educational systems such as different grading systems or teachers’ expectations, as well as academic motivation of students within and between nations that may differentially influence self-regulation and its relation with academic performance. Therefore, we view this study only as a starting point for investigating the impact of independent and interdependent cultures on the relation of self-regulation and math performance. Future studies conducted in other independent or interdependent cultures should clarify whether the observed results are really due to this cultural difference or to other educational or cultural differences, which are particular to the specific countries studied here. Second, German students of Human Sciences were offered different reimbursement than other participants since the study in which they participated, was part of a larger project consisting of 4-h experiment. Hence, we acknowledge that different incentives in German students of Human Sciences in comparison to other participants might generate participation bias and account partially for the findings of the current study. Third, self-regulation consists of several components such as cognitive, behavioral, and emotional aspects that are differentially related to mathematics performance and their effects should be investigated individually in the future research. Forth limitation is the small sample size of the present study that may preclude a definitive statement for the present study. The last, but not least, important limitation is construct validity in the present study, as our research measurement for assessing self-regulation was designed and validated for Western countries. The problem is that in self-reports, participants of one cultural context may interpret the words differently and compare themselves with different standards than those in another cultural context (e.g., Heine et al., 2002). In our study, the internal consistency of the self-regulation self-report in Iranian students is sufficient for the present study and in line with previously reported findings in Eastern countries such as China (Cronbach’s α = 0.75; Unger et al., 2016), however, it should be also noted that it is relatively low, which can be due to either a reliability or homogeneity problem. In the future, international researchers should strive for a transcultural self-regulation scale, which can be used in Western and non-Western cultures with high reliability and validity.

## Conclusion

In conclusion, our findings show that the relation of self-regulation and mathematics performance is similar in German and Iranian college students. In addition, the effect of field of study on the relation of self-regulation and mathematics performance was highlighted in the present study. Self-regulation did not predict mathematics performance in German and Iranian students, however, when the effect of field of study was taken into account, self-regulation predicted mathematics performance in students of less math-related fields of study within each country. It is important to note that while the single analysis produced differential results, a direct comparison of the different fields of studies was non-significant – therefore, we have interpreted these results with great care. Nevertheless, since the relation between self-regulation and mathematics performance within each country, was significant only for less math-related fields of study, we suggest that the possible confounding effect of field of study should be considered in studies when the relation of self-regulation and mathematics performance is examined.

## Data Availability Statement

The datasets generated for this study are available on request to the corresponding author.

## Ethics Statement

Ethical review and approval was not required for the study on human participants in accordance with the local legislation and institutional requirements. The patients/participants provided their written informed consent to participate in this study.

## Author Contributions

PN and CG designed and performed the research. PN and JK analyzed the data. PN, JK, CG, and H-CN wrote the manuscript. All authors contributed to the article and approved the submitted version.

## Conflict of Interest

The authors declare that the research was conducted in the absence of any commercial or financial relationships that could be construed as a potential conflict of interest.

## Footnotes

1 This study used part of the data of the German participants that was published by Nemati et al. (2017).

2 Descriptive statistics for each subsample field of study (i.e., German and Iranian students of Human Sciences and Engineering\Informatics) and the correlation matrix in German and Iranian students are presented in Appendices B, C, respectively for the interested reader.

## Appendix A

### Ceiling/Floor Effect for Multiplication ER

**TABLE:**

**Sample**	***n***	***M (SD)***	***K-S^*a*^***	***Skewness***	***Kurtosis***
German students	60	0.18 (0.10)	0.077	0.94	1.53
Iranian students	62	0.19 (0.10)	0.001	1.16	1.36

*^*a*^Kolmogorov–Smirnov *p*-values.*

## Appendix B

### Descriptive and Test Statistics of Background Characteristics and Study Measurements in German and Iranian Students of Human Sciences and Engineering/Informatics

**TABLE B1:** Analysis of variance of country and field of study on ER.

**Source**	***df***	***MS***	***F***	***p***	**Partial η^2^**
Country	1	0.14	18.64	**<0.001**	0.14
Field of study	1	0.13	18.52	**<0.001**	0.14
Country × field of study	1	0.13	17.76	**<0.001**	0.13
Error	118	0.01			

*MS, mean squares. Bold *p*-values depict *p* < 0.05.*

**TABLE B2:** Analysis of variance of country and field of study on RT.

**Source**	***df***	***MS***	***F***	***p***	**Partial η^2^**
Country	1	5.61	23.01	**<0.001**	0.16
Field of study	1	6.47	26.60	**<0.001**	0.18
Country × field of study	1	2.71	11.12	**0.001**	0.09
Error	118	0.24			

*MS, mean squares. Bold *p*-values depict *p* < 0.05.*

**TABLE B3:** Descriptive of German students of Human Sciences and Engineering/Informatics.

**Variable**	**Human Sciences**		**Engineering/Informatics**		**Diff**
	***M* (*SD*)**	***K-S^a^***	***M* (*SD*)**	***K-S^a^***	***p***
Age (years)	20.95 (1.08)	0.001	21.55 (1.19)	<0.001	**0.039**^b^
Gender, female (%)	82		40		**0.002**^c^
Math self-concept	2.75 (0.84)	<0.001	2.65 (0.74)	<0.001	0.568^b^
Expectancy of success	2.78 (0.73)	<0.001	3.10 (0.31)	<0.001	0.052^b^
Self-regulation	41.43 (8.74)	0.200	39.90 (8.20)	0.200	0.518^d^
Multiplication performance					
ER	0.19 (0.11)	0.028	0.18 (0.08)	0.200	0.742^b^
RT(s)	3.10 (0.56)	0.200	2.93 (0.48)	0.200	0.259^d^

*^*a*^Kolmogorov–Smirnov *p*-values, ^*b*^Mann–Whitney *U* test,^*c*^Fisher’s Exact Test, ^*d*^*t*-test. Bold *p*-values depict *p* < 0.05.*

**TABLE B4:** Descriptive of Iranian students of Human Sciences and Engineering/Informatics.

**Variable**	**Human Sciences**		**Engineering/Informatics**		**Diff**
	***M* (*SD*)**	***K-S^a^***	***M* (*SD*)**	***K-S^a^***	***p***
Age (years)	20.09 (1.20)	<0.001	21.00 (0.98)	0.004	**0.001**^b^
Gender, female (%)	50		40		0.456^c^
Math self-concept	1.91 (1.28)	<0.001	3.03 (0.70)	<0.001	**<0.001**^b^
Expectancy of success	2.88 (1.01)	<0.001	3.67 (0.37)	<0.001	**<0.001**^b^
Self-regulation	42.69 (6.57)	0.200	42.43 (6.33)	0.200	0.877^d^
**Multiplication performance**					
ER	0.18 (0.09)	0.151	0.05 (0.04)	<0.001	**<0.001**^b^
RT(s)	2.97 (0.47)	0.007	2.18 (0.42)	0.200	**<0.001**^b^

*^*a*^Kolmogorov–Smirnov *p*-values, ^*b*^Mann–Whitney *U* test,^*C*^Fisher’s Exact Test, ^*d*^*t*-test. Bold *p*-values depict *p* < 0.05.*

## Appendix C

### Correlation Matrix in German and Iranian Students

**TABLE C1:** Correlations between background variables, self-regulation, and multiplication RT in German students.

**Variable**	**1**	**2**	**3**	**4**	**5**	**6**
1. Age	–					
2. Gender	0.29*	–				
3. Math self-concept	−0.21	0.20	–			
4. Expectancy of success	−0.21	0.24	0.43*	–		
5. Self-regulation	−0.14	−0.26*	−0.05	0.07	–	
6. Multiplication RT(s)	0.01	−0.18	−0.11	−0.28*	−0.25	–

*n = 60. * Correlation is significant at the 0.05 level (2-tailed).*

**TABLE C2:** Correlations between background variables, self-regulation, and multiplication RT in Iranian students.

**Variable**	**1**	**2**	**3**	**4**	**5**	**6**
1. Age	–					
2. Gender	0.22	–				
3. Math self-concept	0.29*	0.14	–			
4. Expectancy of success	0.13	0.01	0.34*	–		
5. Self-regulation	0.12	0.10	0.13	−0.17	–	
6. Multiplication RT(s)	−0.11	−0.03	−0.61*	−0.58*	−0.09	–

*n = 62. * Correlation is significant at the 0.05 level (2-tailed).*

## Appendix D

### Linear Model of Age and Gender as Predictors of Multiplication RT

**TABLE D1:** Linear model of predictors of multiplication RT.

**Predictor**	***b***	***SE(B)***	***t***	***p***
Constant	−0.00	0.09	−0.04	0.964
Self-regulation	−0.19	0.09	−2.16	**0.032**
Age	0.04	0.09	0.41	0.684
Self-regulation × age	−0.08	0.09	−0.90	0.372

*N = 122. All variables are standardized. *R*^2^ = 0.05, *F*(3,118) = 2.05, *p* = 0.111. Bold *p*-values depict *p* < 0.05.*

**TABLE D2:** Linear model of predictors of multiplication RT.

**Predictor**	***b***	***SE(B)***	***t***	***p***
Constant	−0.40	0.12	−3.23	**0.002**
Age	−0.10	0.12	−0.86	0.389
Country	0.78	0.17	4.43	**0.000**
Age × country	0.11	0.18	0.63	0.527

*N = 122. All variables are standardized and country was dummy coded. *R*^2^ = 0.15, *F*(3,118) = 6.81, *p* < 0.001. Bold *p*-values depict *p* < 0.05.*

**TABLE D3:** Linear model of predictors of multiplication RT.

**Predictor**	***b***	***SE(B)***	***t***	***p***
Constant	0.17	0.12	1.48	0.142
Self-regulation	−0.28	0.12	−2.39	0.019
Gender	−0.39	0.18	−2.18	0.031
Self-regulation × gender	0.14	0.18	0.81	0.419

*N = 122. All variables are standardized and Gender was dummy coded. *R*^2^ = 0.08, *F*(3,118) = 3.61, *p* = 0.015.*

## Appendix E

### Linear Model of Self-Regulation as the Predictors of Multiplication RT in Students of Human Sciences

**TABLE E1:** Linear model of predictors of multiplication RT in students of Human Sciences.

**Predictor**	***b***	***SE(B)***	***t***	***p***
Constant	0.29	0.15	2.01	**0.048**
Self-regulation	−0.33	0.17	−1.98	0.051
Country	0.17	0.19	0.87	0.389
Self-regulation × country	0.06	0.20	0.29	0.773

*n = 72. All variables are standardized. *R*^2^ = 0.14, *F*(3,68) = 3.75, *p* = 0.015. Bold *p*-values depict *p* < 0.05.*
